# PAGE4 promotes prostate cancer cells survive under oxidative stress through modulating MAPK/JNK/ERK pathway

**DOI:** 10.1186/s13046-019-1032-3

**Published:** 2019-01-18

**Authors:** Chengcheng Lv, Shui Fu, Qingzhuo Dong, Zi Yu, Gejun Zhang, Chuize Kong, Cheng Fu, Yu Zeng

**Affiliations:** 1 Department of Urology, Cancer Hospital of China Medical University, Liaoning Cancer Hospital and Institute, 44 Xiaoheyan Road, Shenyang, 110042 Liaoning China; 2 Department of Urology, The First Hospital of China Medical University, 155 Nanjing North Road, Shenyang, 110001 Liaoning China

**Keywords:** PAGE4, Oxidative stress, Prostate Cancer, MAPK, ERK

## Abstract

**Background:**

Prostate cancer (PCa) is one of the most common cancers in male worldwide. Oxidative stress has been recognized as one of the driving signals pathologically linked to PCa progression. Nevertheless, the association of oxidative stress with PCa progression remains unclear.

**Methods:**

Western blot, q-RT-PCR and bioinformatics analyses were used to examine PAGE4 expression. Comet assay and Annexin V/ PI dual staining assay were performed to investigate DNA damage and cell death under oxidative stress. Mouse xenograft model of PCa cells was established to verify the role of PAGE4 in vivo. Transcriptomic analysis was performed to investigate the underlying mechanism for the function of PAGE4 under oxidative stress. Western blot assay was conducted to determine the status of MAPK pathway. Immunohistochemistry was used to identify protein expression of PAGE4 in tumor tissues.

**Results:**

In this study, we found that PAGE4 expression was increased in PCa cells under oxidative stress condition. PAGE4 overexpression protected PCa cells from oxidative stress-inducing cell death by reducing DNA damage. PAGE4 overexpression promoted PCa cells growth in vivo. Mechanistically, PAGE4 promoted the survival of prostate cancer cells through regulating MAPK pathway which reflected in decreasing the phosphorylation of MAP2K4, JNK and c-JUN but increasing phosphorylation of ERK1/2.

**Conclusion:**

Our findings indicate that PAGE4 protects PCa cells from DNA damage and apoptosis under oxidative stress by modulating MAPK signalling pathway. PAGE4 expression may serve as a prognostic biomarker for clinical applications.

**Electronic supplementary material:**

The online version of this article (10.1186/s13046-019-1032-3) contains supplementary material, which is available to authorized users.

## Background

Prostate cancer (PCa) remains the third killer in men with all kinds of cancers [1]. Although the 5-year survival rate for most men with local or regional prostate cancer is almost 100%, for those patients with tumors spreading to other parts of the body, the 5-year survival rate is merely 29% [1]. Basically, androgen deprivation therapy can delay the progression of cancer; however, many patients would end up with castration resistant prostate cancer (CRPC), which is currently incurable [2].

PAGE4 (Prostate Associate Gene 4) belongs to the Cancer Testis Antigen (CTA) family, which are normally expressed only in adult testis but not in other mature tissues. However, in many tumors or other diseases, the CTA genes are re-expressed again [3, 4]. Accumulating evidence from varied studies including those from our group has shown that PAGE4 is re-expressed in human diseased prostate, particularly in the stroma of symptomatic benign prostatic hyperplasia (BPH) [5], and in the epithelia of proliferative inflammatory atrophy (PIA) that is considered as a precursor of PCa, as well as in PCa itself [6–11]. Interestingly, the expression of PAGE4 was found to be significantly lower in androgen-resistant prostate cancer than primary prostate cancer [8]. Additionally, PAGE4 was recently found to be related to androgen receptor function, while different phosphorylated format catalyzed by two kinases contributed to PAGE4 conformational dynamics and androgen-dependence switching in PCa [12, 13]. We previously screened prognostic indicators of prostate cancer using tissue samples, and found that the transcription level of PAGE4 in prostate cancer tissues was negatively correlated with the biological recurrence of cancer [14]. At the same time, another study indicated that the expression level of PAGE4 varied in prostate cancer tissues with different Gleason scores [15], suggesting that it might be used as an auxiliary indicator of prostate cancer malignancy. Thus, the involvement of PAGE4 in diseased prostate, particularly in the prostate cancer, is somehow founded. However, the exact functional role of PAGE4 in the context of signalling pathways under a diseased status of prostate cancer remains largely unclear.

Our previous study has found that PAGE4 is an intrinsic disorder protein (IDP) and can protect cells from stress [16, 17]. Further studies have shown that the regulatory mechanism of PAGE4 may be related to the phosphorylation of the Thr-51 amino acid site stimulated by the homeodomain-interacting protein kinase 1 (HIPK1) [18, 19] and of multiple S/T residues catalyzed by CDC-Like Kinase 2 (CLK2) [13]. In cellular process, we previously showed that PAGE4 mainly localized in mitochondria, and overexpressing PAGE4 protected cancer cells from different kinds of stress such as glucose deprivation, treatment of TNFα or chemical drug, through increasing the protein level of p21 [10]. However, when cells were exposed to H_2_O_2_, p21 level was not changed, suggesting that PAGE4 may protect cells from oxidative stress in a different manner. One possible explanation of this phenomenon is that PAGE4 interacts with c-JUN and then activates MAP kinase signalling pathway [20], but the detail mechanism still needs to be further studied.

Here, we focused on the PAGE4 regulation on oxidative stress in PCa cells. We found that treatment of H_2_O_2_ significantly increased the expression of PAGE4, which exactly protected cells from apoptosis under the stress status. In addition, PAGE4 overexpression promoted tumor growth in xenograft mouse models. Further RNA-sequencing study revealed a potential involvement of Mitogen-activated protein kinase (MAPK) pathway in PAGE4 regulation on oxidative stress. Thus, the present results may expand our understanding of the role of PAGE4 on stress response to reactive oxygen species (ROS) which is critical to the development of PCa.

## Methods

### Cell culture

All cell lines were obtained from the Cell Bank of the Shanghai Institutes of Biological Sciences, Chinese Academy of Sciences. Human prostate cancer cell lines 22RV1, DU145, PC3 and LNCaP were cultured in RPMI-1640 (Gibco, China), 293 T cells were cultured in DMEM (Gibco, China), and both media contained 10% fetal bovine serum (Gemini, USA) and 1% penicillin-streptomycin (Gibco, USA). All cell lines were cultured at 37 °C in a humidified atmosphere of 5% CO_2_.

### Construction and production of lentiviral vector

Open reading frame of PAGE4 with HA N-terminal tag was cloned into pLVX-TetOne-Puro (Clontech, USA) vector using In-Fusion HD method following the protocol provided by Clontech. Briefly, vector was digested by EcoR I (New England Biolabs, USA) and BamH I for linearization, then mixed with insert DNAs in In-Fusion cloning reaction buffer. Alternatively, open reading frame of CLK2 or HIPK1 with 3X Flag N-terminal tag were amplified from human cDNA library, and then digested by BamH I and Xba I for CLK2, and Xho I and Not I for HIPK1. The insert DNAs were cloned into pLVX-Puro (Clontech) vector. After construction, vectors were transduced into *E. coli*. Screened clones were expanded, and plasmids were extracted and purified. Then the over-expressing vectors or empty vectors were co-transfected with 2nd Generation Packaging System Mix (Abmgood, Canada) into 293 T cells using calcium phosphate transfection method [21]. After culturing transfected 293 T cells in DMEM media for 48 h, the supernatant containing lentivirus were harvested and filtered with a 0.45 μm filter (Merck Millipore, Germany).

### Cell transfection

Lentivirus were added into target cell lines with 8 μg/mL polybrene (Sigma-Aldrich, USA), then cells were centrifuged for 1 h at 500 rpm to aid transfection. After 72 h, cells were treated with 2 μg/mL of puromycin (Invivogen, USA) for 2 weeks, and survival cells were maintained in medium supplemented with 200 ng/mL of puromycin.

### Cell treatment

To enable the expression of transduced gene, 1 mg/L of doxycycline (DOX, Clontech) was added into all transfected cells for at least 48 h before analysis. To stimulate ROS in vitro, 200 mM H_2_O_2_ was added to cells for 12 h before analysis unless otherwise stated. 5 mM N-Acetyl-L-cysteine (NAC, Sigma-Aldrich) was given to cells for 12 h before collection.

### RNA extraction and real-time quantitative PCR

Total RNA was extracted using TRIzol reagent (Invitrogen, USA) according to the manufacturer’s instructions. cDNA was synthesized using PrimeScript™ RT Master Mix (Takara, Japan), and quantitative real-time PCR was performed using SYBR Premix EX Taq™ II (Takara). ACTB was used as a reference gene. The primer sequences used to amplify the target genes are searched from PrimerBank [22, 23]. The sequence of primers are listed in Additional file 1: Table S1. The real-time PCR was performed as follows: 3 μL of ddH_2_O, 5 μL of 2× SYBR mix, 0.5 μL of forward primer, 0.5 μL of reverse primer and 1 μL cDNA templates were mixed together. The PCR reaction consisted of an initial denaturation at 95 °C for 30 s, followed by 40 cycles of denaturation at 95 °C for 5 s, annealing at 60 °C for 30 s. The ΔCT method was performed to calculate the relative expression [24].

### ROS assay

ROS activity was measured with 2′, 7′ –dichlorofluorescin diacetate (DCFDA, Sigma). Cells were seeded at 25,000 cells per wells in 96 well plates the day before assay. When experiment, cells were washed with PBS once, then stained with 20 μM of DCFDA for 30 min for LNCaP cells, and 25 μM of DCFDA for 45 min for DU145 cells, respectively. After washed with PBS, cells were treated with 2 mM of H_2_O_2_ or PBS in DMEM medium without supplemental phenol red for one hour, then were measured under microplate reader (BioTek Instruments, USA). Unstained cells from each group were used for standardization.

### Western blotting

Cells were harvested in RIPA lysis buffer (Beyotime, China), then the protein concentrations were measured using the BCA protein assay kit (Invitrogen, USA). After that, protein was heated for 5 min at 99 °C, and 20 μg of protein were loaded into each lane and separated by 10% SDS-PAGE in Tris-glycine running buffer, and then transferred to PVDF membranes (Millipore). The membranes were blocked with 5% skimmed milk in TBST at room temperature for 1 h and then incubated with primary antibodies at 4 °C overnight. After three washes with TBST for 5 min each, the membranes were incubated with 1:3000 HRP-coupled secondary antibody (Abmgood, Canada) in 5% skimmed milk in TBST for 1 h. The membranes were then washed for three more times with TBST and visualized using the Pierce ECL Plus (Thermofisher, USA). Antibodies against PAGE4 (Sigma-Aldrich, USA) was diluted in 5% skimmed milk in TBST at 1:500, and antibody against β-actin (Sigma-Aldrich) was diluted in 5% skimmed milk in TBST at 1:5000, while antibodies against pMAP2K4, pJNK, JNK, pATF2, p-c-JUN, pERK, ERK (Cell Signaling Technology, USA) were all diluted in 5% bovine serum albumin (Sangon, China) in TBST at 1:1000. Integral optical density (IOD) of each band was measured by using Gel-Pro Analyzer (version 4.0, Media Cybernetics, USA).

### Comet assay

The alkaline comet assay was performed using CometAssay Reagent kit for Single Cell Gel Electrophoresis Assay (Trevigen, USA) according to the manufacturer’s protocol. Briefly, cells were collected then suspended at 1 × 10^5^ cells per mL, and 20 μL cell suspension combined with 200 μL LMAgarose with final volume of 50 μL were then pipetted onto each CometSlide. When a clear ring appeared, the slides were incubated in Lysis Solution for 30 min. Then the slides were sunk in alkaline unwinding solution for 20 min at room temperature. Slides were electrophoresed in alkaline electrophoresis solution at 21 V, 300 mA for 30 min at 4 °C. The slides were drained and stained with 1× SYBR Gold (ThermoFisher). Cell images were captured using an Olympus AX70 fluorescence microscope (Olympus, Japan) and single cells tail length, tail DNA percentage were measured by CometScore version 2.0 (TriTek, USA) software [25].

### Annexin V/ Propidium iodide (PI) double-staining assay

Annexin V-FITC apoptosis detection kit (BD biosciences, USA) was used to analyse the apoptosis. Annexin V and propidium iodide staining was performed according to the manufacturer’s protocol. Briefly, 10^4^ cells in 100 μL 1× Binding Buffer were stained with 5 μL FITC Annexin V and 5 μL PI for 15 min at room temperature. Stained cells were detected using a FACScan flow cytometer (BD biosciences) and data was collected using FlowJo version 10.0.7 (FlowJo, USA) software. FITC positive cells whether or not with PI positive staining were calculated as apoptotic cells.

### Xenograft tumor model

Twelve severe combined immunodeficiency (SCID) mice with 7 weeks old were randomly divided into three groups, 4 in each. DU145 cells (2.5 × 10^6^) that were stably transfected with conditional PAGE4-overexpressing vector or empty vector were mixed with matrigel (Coring, USA) at a ratio of 1:1, and then injected subcutaneously in both flanks of mice in each group under isoflurane anaesthesia [26]. DOX was added in drinking water at 500 mg/L with 3% sucrose to all mice [27]. Normal saline or 200 mg/kg NAC was intraperitoneal injected daily from day14 [28]. At day 28, the animals were sacrificed and the tumors were removed, weighed and homogenized to extract RNA.

### RNA-sequencing and analysis

RNA was extracted from different cell lines using RNeasy Plus Universal Kits (QIAGEN, Germany) according to the manufacturer’s instructions. RNA library construction and sequencing was performed by Berry Genomics (Beijing). Raw RNA sequences data were then mapped to the GRCh38 (hg38) genome using HISAT2 2.1.0 [29]. FeatureCounts 1.6.0 [30] was used to count reads and generate raw counts for per gene. Differential analysis of count data was carried out by DESeq2 3.7 [31]. Differentially expressed genes (DEGs) were then identified using fold change of the target group to the control group ratios > 1.5. Gene Set Enrichment Analysis (GSEA) [32] was performed by using publicly available software downloaded from the Broad Institute [33]. Main parameters setting of GSEA were weighted enrichment statistic and Diff_of_Classes metric for ranking genes. Protein-protein interaction (PPI) was analysed through String [34]. Sequencing data have been deposited in Gene Expression Omnibus (GEO) with accession number GSE119005.

### Dataset analysis

The TCGA-PRAD data was downloaded from the TCGA portal [35]. This dataset includes the RNA sequencing data of 498 tumor tissue samples, generated using Ilumina sequencing technology. And R packages for Survival Analysis [36] was used to perform disease-free survival analysis. 109 samples were defined as PAGE4 high expression and 84 samples were low expression.

### Immunohistochemistry

Expression of PAGE4 and pERK1/2 was assessed by immunohistochemistry using paraffin-embedded tissue sections obtained from the department of pathology in Cancer Hospital of China Medical University. Briefly, the sections were deparaffinised and then boiled in citrate unmasking solution for 35 min. After incubated in 3% hydrogen peroxide for 10 min, sections were blocked with 5% BSA for 1 h at room temperature, and then incubated with rabbit anti-human PAGE4 (1:200, Abcam, USA) or rabbit anti-human pERK1/2 (1:400, CST, USA) antibodies overnight at 4 °C. After that, sections were incubated with appropriate secondary antibodies (Zsbio, China) and then stained with DAB & Hematoxylin.

### Statistical analysis

All in vitro experiments were repeated at least three times. Data was shown as mean ± SEM, and statistical analyses were carried out using SPSS 22.0 (IBM, USA) or Graphpad Prism 7 (Graphpad, USA). The Student’s t-test was used to evaluate the significance of differences for Comet assay, Annexin V/ Propidium Iodide (PI) double-staining assay and ROS assay. Mann-Whitney U test was used to evaluate the significance of differences for q-RT-PCR, Western blot and tumor weight. For survival analysis, the Kaplan-Meier method was used to estimate the survival curves, and the log-rank test for the comparison. A *p*-value < 0.05 was considered significant.

## Results

### Expression level of PAGE4 was increased under ROS stimulation in prostate cancer cell lines

**Fig. 1:**
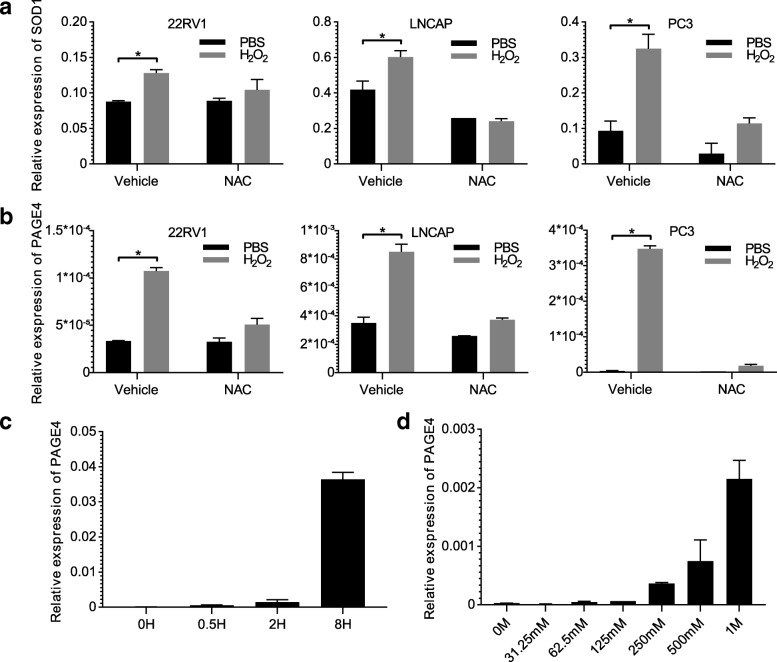
Expression of PAGE4 when exposed to H_2_O_2_ in prostate cancer cells. mRNA expression of SOD1 (**a**) and PAGE4 **(b)** in 22RV1, LNCaP and PC3 cells, which were treated with 2 mM H_2_O_2_ combined with or without 5 mM NAC for 12 h, *P < 0.05. **c** mRNA expression of PAGE4 in 22RV1 cells treated with 50 mM H_2_O_2_ for 0, 0.5, 2, 8 h. **d** mRNA expression of PAGE4 in 22RV1 cells treated with different dosages of H_2_O_2_ for 12 h. All experiments were repeated at least 3 times

ROS can induce DNA damage and increase the risk of DNA mutation that may lead to activation of oncogene and development of cancer [37, 38]. Knowing how ROS acts in prostate cancer can help us find ways to fight against cancer. To test, we used H_2_O_2_ to simulate ROS in prostate cancer cell lines 22RV1, DU145, PC3, and LNCaP, which cover both androgen-sensitive and –insensitive cell lines. As shown in Fig. 1 a, ROS related gene SOD1 was increased after H_2_O_2_ treatment in all the cell lines, suggesting that prostate cancer cells were indeed experienced a high level of oxidative stress no matter a subtype of PCa cells. In addition, endogenous ROS assay also showed that cells tolerated a high oxidative stress after hydrogen peroxide treatment (Additional file 2: Figure S1). At the same time, NAC that is a penetrating antioxidant significantly reduced the oxidative stress caused by H_2_O_2_ in cells, indicated by reducing the transcriptional level of SOD1 (Fig. 1 a). On the other hand, the mRNA level of PAGE4 was increased in all of these lines after H_2_O_2_ treatment (Fig. 1 b), while NAC treatment effectively inhibited the effect of H_2_O_2_ on PAGE4 expression. Moreover, the upregulation of PAGE4 under H_2_O_2_ treatment showed a typical manner of time-dependent and dose-dependent (Fig. 1 c, d). Accordingly, in 22RV1 cells, the expression of PAGE4 was greatly increased when cells were treated with H_2_O_2_ from 0 h to 8 h. Similarly, more H_2_O_2_ was added, higher levels of PAGE4 mRNA were detected. These results suggest that the mRNA expression of PAGE4 be induced by ROS stress.

### Prostate cancer cells with PAGE4 overexpression tolerate oxidative stress

**Fig. 2:**
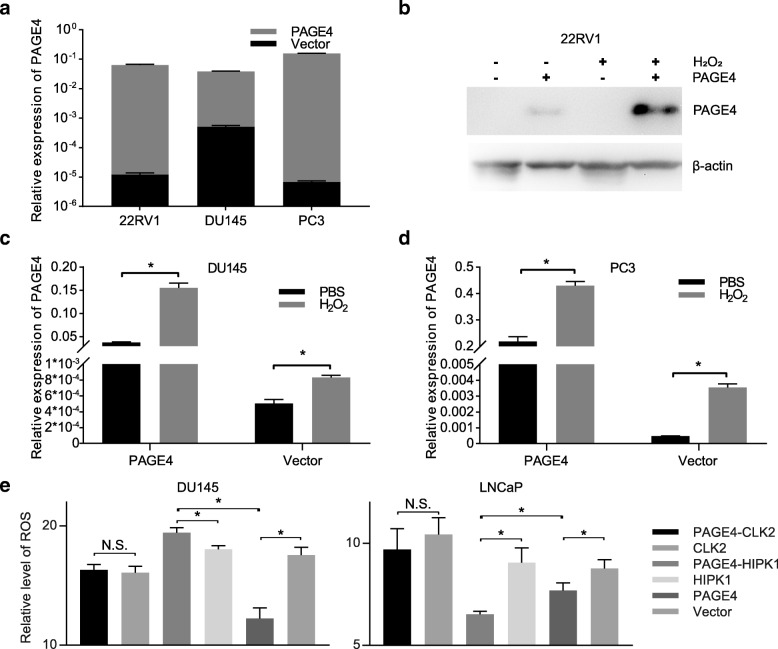
Overexpression of PAGE4 in PCa cells. **a** mRNA expression of PAGE4 in 22RV1, DU145 and PC3 cells after transfected with conditional overexpressing-vector of PAGE4. **b** Protein expression of PAGE4 in PAGE4-transfected 22RV1 cells with or without treatment of H_2_O_2_. **c d** mRNA expression of PAGE4 in PAGE4-transfected DU145 and PC3 cells after H_2_O_2_ stimulation, *P < 0.05. **e** DU145 and LNCaP cells were co-transfected PAGE4 with either CLK2 or HIPK1. Cells were treated with H_2_O_2_ stimulation, and the intracellular ROS were measured, *P < 0.05. All experiments were repeated at least 3 times

We have previously shown that silencing PAGE4 expression inhibits cell survival and enhances chemo-cytotoxicity in prostate cancer cells [16]. In such circumstance, PAGE4 may protect cancer cells through upregulation of p21 in most stress status with exception of H_2_O_2_, in which p21 level was not altered in either PAGE4-expressing cells or PAGE4-knocking down cells [10]. To investigate how PAGE4 is involved in oxidative stress protection in prostate cancer, we established inducible PAGE4-overexpressing cell lines, in which both mRNA and protein levels of PAGE4 were significantly increased by treatment of DOX (Fig. 2 a, b). As shown in Fig. 2 b, c, d, the expression of PAGE4 were induced by H_2_O_2_ both in empty vector and PAGE4 construct-transfected cells, even though the basic mRNA level of PAGE4 was much higher in PAGE4 construct-transfected cells than empty vector-transfected cells. The fact that both endogenous and exogenous PAGE4 gene can be induced by ROS suggests that the regulation of PAGE4 by ROS may be related to a post-transcriptional way.

Previous studies demonstrated that CLK2 and HIPK1 both phosphorylate PAGE4 and therefore differently regulate its activity [13, 39]. We ask whether the specific phosphorylation state of PAGE4 impact its regulation in ROS response. To test, CLK2 or HIPK1 were co-transfected with PAGE4 into DU145 or LNCaP cells and treated with H_2_O_2_. Then, the intracellular levels of ROS were measured to evaluate the oxidative stress status. As shown in Fig. 2e, while PAGE4 overexpression alone reduced ROS production in both cell lines, co-transfecting PAGE4 with CLK2 did not impact ROS level compared to control cells. This result suggests that CLK2 inhibit PAGE4 activity in suppressing ROS production under ROS stress. However, when PAGE4 was co-tranfected with HIPK1, the intracellular ROS was changed differently in these two cell lines. Namely, in LNCaP cells, co-expression of HIPK1 with PAGE4 reduced ROS production even less than what caused by overexpression of PAGE4 alone after H_2_O_2_ treatment. By contrast, in DU145 cells, co-transfection of HIPK1 and PAGE4 increased but not decreased ROS level as compared to control cells. These findings suggest that HIPK1 may impact PAGE4 function during ROS response in a cell type-dependent manner, although further study is clearly needed.

**Fig. 3:**
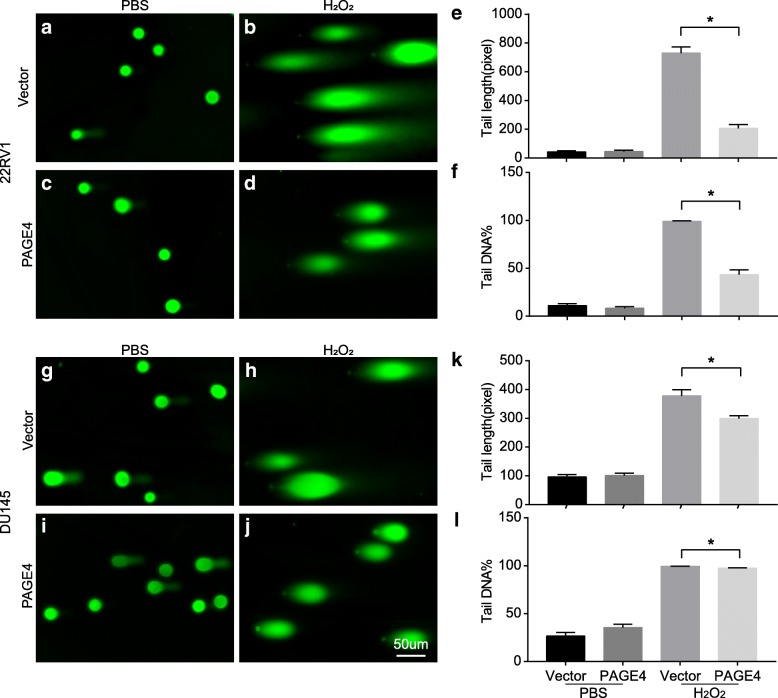
Alkaline comet assay for PAGE4 overexpressed cells when exposed to H_2_O_2_. **a**-**d** Representative images of comets assay in 22RV1 cells that were transfected with either PAGE4 or empty vector and treated with PBS or H_2_O_2_. **e** The statistical analysis of tail length. **f** The statistical analysis of tail DNA percentage, *P < 0.05. **g**-**j** Representative images of comets assay in DU145 cells that were transfected with either PAGE4 or empty vector and treated with PBS or H_2_O_2_. **k** The statistical analysis of tail length. **l** The statistical analysis of tail DNA percentage, *P < 0.05. All experiments were repeated 3 times and at least 30 cells per group were counted

**Fig. 4:**
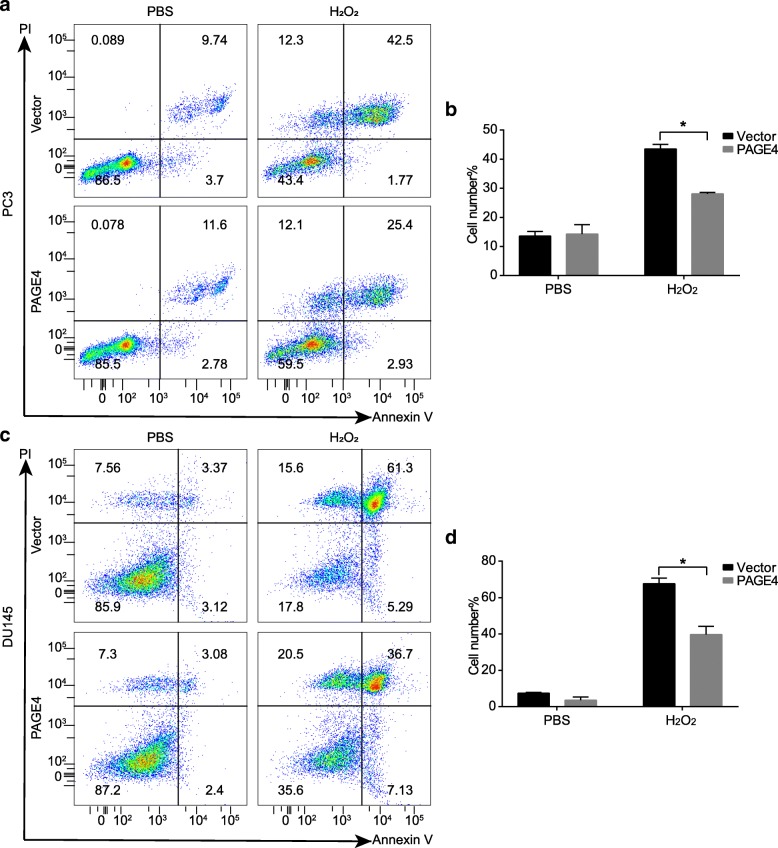
Apoptosis analysis for PAGE4 overexpressing cells when exposed to H_2_O_2_. **a** PC3 cells with or without PAGE4 overexpression were stained with Annexin V/PI. The percentage of apoptotic cells was calculated using flow cytometry. **b** The statistical analysis of apoptosis assay results, *P < 0.05. **c** DU145 cells with or without PAGE4 overexpression were stained with Annexin V/PI. The percentage of apoptotic cells was calculated using flow cytometry. **d** The statistical analysis of apoptosis assay results, *P < 0.05. Experiments were repeated 3 times

Next, to test DNA damage, a comet assay was performed in DU145 and PC3 cell lines with or without PAGE4 overexpression after exposed to H_2_O_2_ for 12 h. As shown in Fig. 3, a lower level of DNA damage was found in PAGE4 overexpressing cell lines when compared with empty vector-transfected cell lines, indicated by shorter tails and smaller tail DNA percentage in PAGE4 overexpressing cell lines. At the same time, in a flow cytometry, PAGE4 overexpressing cell lines also showed a remarkable low level of apoptosis when exposed to H_2_O_2_ compared to empty vector-transfected cell lines (Fig. 4). Above all, the overexpression of PAGE4 in prostate cancer cell lines can protect cancer cells from cell death that is caused by oxidative stress through reducing DNA damage.

### Overexpressing PAGE4 promotes tumor growth in vivo

**Fig. 5:**
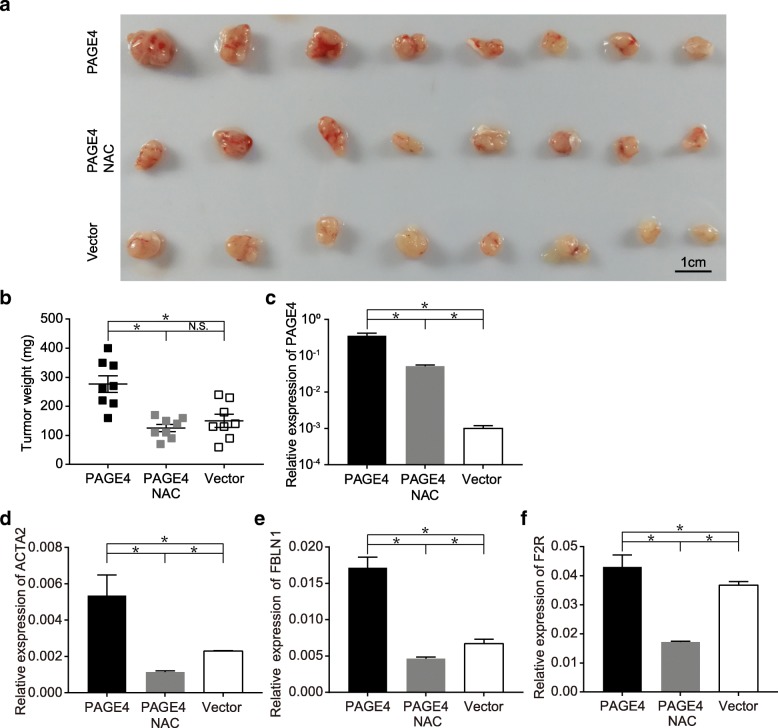
PAGE4 promotes prostate cancer cell growth but reduce tumor malignancy in vivo. **a** Tumor formation of DU145 cells with or without PAGE4 overexpression. Mice were feed with DOX in drinking water and NAC was administered by intraperitoneal injection. **b** Tumor weight in different groups, *n* = 8 in each group, *P < 0.05. **c** Expression of PAGE4 in xenografted tumor tissues was measured with Q-PCR, *P < 0.05. **d** Expression of ACTA2 in xenografted tumor tissues was measured with Q-PCR, *P < 0.05. **e** Expression of FBLN1 in xenografted tumor tissues was measured with Q-PCR, *P < 0.05. **f** Expression of F2R in xenografted tumor tissues was measured with Q-PCR, *P < 0.05. Three tumors were randomly picked and subjected to Q-PCR assay

Having known that PAGE4 can protect prostate cancer cells from oxidative stress in vitro, we further investigated whether PAGE4 promotes tumor growth in vivo. As shown in Fig. 5 a, b, PAGE4-overexpressing cells formed larger tumors in SCID mice than cells without PAGE4 overexpression, while the body weight of mice were not much different between three groups during the whole experiment (Additional file 3: Figure S2). These results suggest that PAGE4 overexpression in vivo indeed enhances tumor growth in mice. Given that proliferating cancer cells may suffer a high oxidative stress in vivo, we ask whether ROS level in the body of mice affect tumor growth. When we treated the mice with antioxidant NAC, we found that the promoting effect of PAGE4 overexpression on tumor growth was partially impeded (Fig. 5 a, b). At the same time, in consistence with the above in vitro study (Fig. 1), PAGE4 mRNA expression in tumor tissues was also significantly inhibited by NAC treatment in mice, although the transcription of PAGE4 is largely derived from the exogenous construct, namely PAGE4-overexpressing vector (Fig. 5 c). These results reveal that PAGE4 is an indeed ROS-response gene and can promote tumor growth when induced by ROS stress.

In addition, several genes that were reported to be related to a less cancer aggressiveness or metastatic potential were found to be increased upon PAGE4 overexpression, including ACTA2, FBLN1 and F2R (Fig. 5 d, e, f). This result may suggest that PAGE4 overexpression enhance tumor growth but not cancer aggressiveness.

### PAGE4 regulation under oxidative stress is related to MAPK pathway

**Fig. 6:**
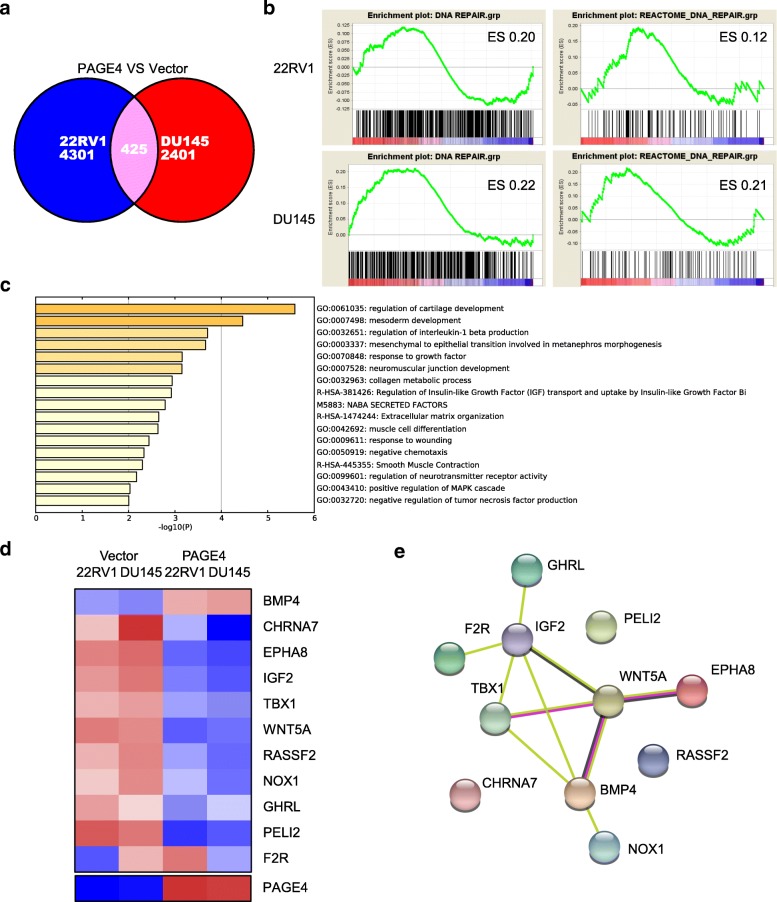
RNA-sequencing and analysis of DEG in prostate cancer cell lines with or without PAGE4 overexpression. **a** Venn diagrams show the number of common and unique DEGs between 22RV1 and DU145 cells. Cells were treated with H_2_O_2_ before RNA sequencing. **b** Signalling pathway enrichment for DEGs. **c** Heat map of Metascape enrichment for DEGs. **d** Heat map of MAPK pathway related genes enriched in Metascape. **e** PPI analysis for MAPK pathway related genes enriched in Metascape

To investigate how PAGE4 is involved in cell survival under oxidative stress, we preformed RNA sequencing in 22RV1 and DU145 cells with or without PAGE4 overexpression after H_2_O_2_ treatment. Totally, 4726 DEGs were found in 22RV1 cells and 2826 DEGs in DU145 cells when comparing those with or without PAGE4 overexpression (Fig. 6 a). Among these DEGs, 425 are shared in both 22RV1 and DU145 cells (Additional file 4: Table S2).

GSEA analysis showed that these DEGs were enriched in the pathways involved in DNA damage repair (Fig. 6 b). DNA damage and repair genes were previously reported to impact the metastatic potential of prostate cancer cells [40], here we found that cells overexpressing PAGE4 exhibited a gene expression pattern somehow of a low metastatic potential (Additional file 5: Figure S3a). In support, by analysing TCGA data, we found that patients with high expression of PAGE4 in tumor tissues had longer disease-free survival (DFS) time than those with low PAGE4 expression (Additional file 5: Figure S3b).

Next, we conducted Metascape [41] pathway analysis [42] on 425 DEGs and found that the enriched pathways can be clustered in 17 groups (Fig. 6 c). Further, we focused on genes enriched in positive regulation of MAPK cascade, namely BMP4, CHRNA7, EPHA8, F2R, IGF2, TBX1, WNT5A, RASSF2, NOX1, GHRL and PELI2 (Fig. 6 d, e).

### PAGE4 regulates MAPK pathway

**Fig. 7:**
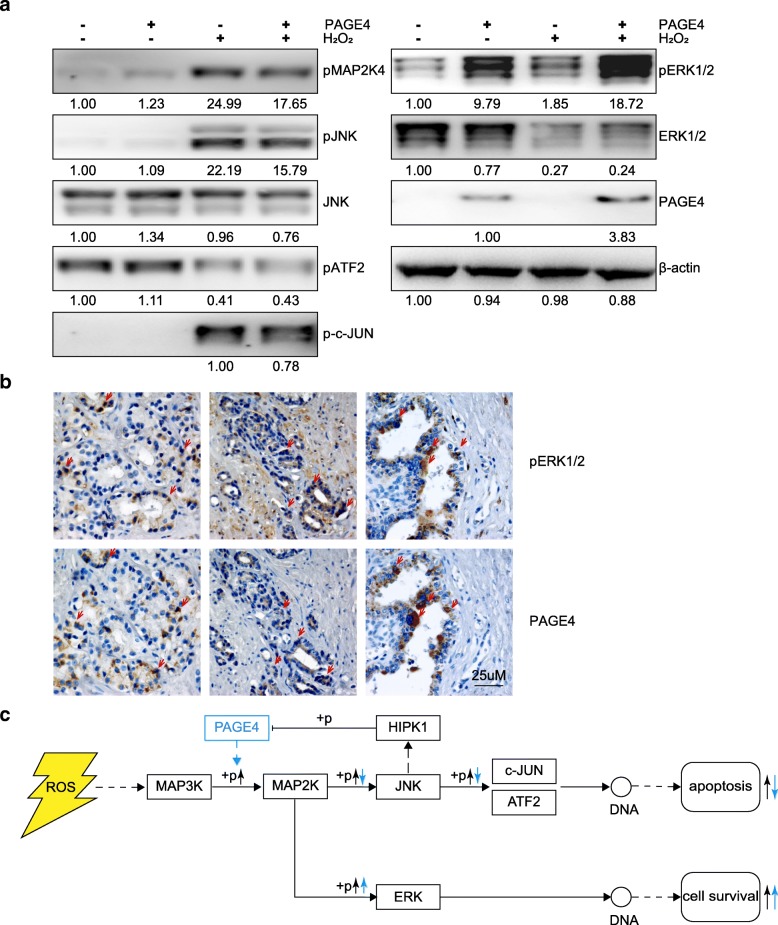
MAPK pathway changed when exposed to H_2_O_2_ in PAGE4 overexpressed cell lines. **a** Expression of MAPK pathway associated proteins in PAGE4 overexpressing cell lines with or without H_2_O_2_ treatment. Integral optical density (IOD) of each band were measured by using Gel-Pro Analyzer. **b** Immunohistochemical staining of pERK1/2 and PAGE4 in human prostate cancer tissues, red arrows showing a likely collocation of two proteins. **c** Hypothesis on PAGE4 regulating MAPK pathway under oxidative stress in prostate cancer cells

Based on the results of RNA sequencing, we next explored how genes in MAPK pathway are regulated by PAGE4 under oxidative stress. When exposed to H_2_O_2_, all cell lines showed activation of MAPK pathway marked by increased phosphorylation of MAP2K4, JNK, and c-JUN but decreased phosphorylation of ATF2, suggesting that oxidative stress effectively induce MAPK pathway signalling in these cells. In cells with PAGE4 overexpression, there was a lower phosphorylation of MAP2K4, JNK and c-JUN than control groups (line 4 comparing to line 3 in Fig. 7 a), suggesting that PAGE4 may reduce cell death through suppressing the activation of JNK.

Another key factor in MAPK pathway is ERK 1/2, which helps cells survive [43, 44]. Here we found that phosphorylated ERK 1/2 was significantly increased in PAGE4 overexpressing cells, no matter whether H_2_O_2_ was added. This may suggest that PAGE4 is involved in ERK 1/2 phosphorylation separately from ROS status. On the other hand, after H_2_O_2_ treatment, total ERK was decreased in both PAGE4 overexpressing and control cells, while phosphorylated ERK 1/2 was increased in both cells, with particular enhancement in PAGE4 overexpressing cells. To some extent, this result may suggest that ERK 1/2 phosphorylation which is likely a survival signalling of cells under ROS stress is much enhanced by PAGE4 overexpression. Thus, elevated of ERK 1/2 phosphorylation induced by overexpression of PAGE4 may be a mechanism of cell survival under oxidative stress. Furthermore, we examined the protein expression of PAGE4 and phosphorylated ERK1/2 in prostate cancer tissues. To support our hypothesis, the two proteins were largely co-expressed in either prostate cancer cells or normal prostate epithelia (Fig. 7 b), suggesting a potential association of PAGE4 function with the activation of MAPK pathway.

Taken together, as shown in Fig. 7 c, it is speculated that oxidative stress induce cell death partly through activating the JNK pathway which has been known to be able to trigger apoptotic cascade. In contrast, ERK that promotes cell survival is also activated during ROS stress to balance the cell death. In this context, PAGE4 protect cells from oxidative stress potentially by reducing cell death message while enhancing cell survival signals.

## Discussion

It is well known that, in the aged prostate where prostate cancer is most often developed, cells are commonly suffered a heavy oxidative stress due to aging [45], smoking [46, 47] as well as obesity [48]. In addition, cancer cells keep proliferating under a hypoxia condition, which itself produces more ROS than normal condition. Apparently, the oxidative stresses existing in the tumor microenvironment is a double-blade sword. On one side, ROS kills cells by inducing apoptosis, while on the other side, ROS remodels cells by causing more harmful DNA mutation [49], which eventually drive an aggressive cancer. For example, ROS in the prostate has been shown to promote epithelial cells transformation [50]. In this scenario, antioxidants or protection of ROS may play a dual role either, in that enhancing cell survival and reducing harmful DNA damage, which may lead to an attenuated cancer evolution towards more aggressive phenotype in a microenvironment enriched with ROS pressure [40, 51, 52].

In the present study, we found that PAGE4 worked exactly in this protective way in PCa cells under oxidative stress status. Firstly, ROS induced PAGE4 expression, which in turn protected cells from apoptosis and DNA damage in vitro and promoted tumor growth in vivo*.* However, when we checked the expression of several tumor less aggressiveness-related genes, such as ACTA2 [53], FBLN1 [54], F2R [55], we found that the expressions of these genes were increased upon overexpression of PAGE4. In addition, RNA sequencing data confirmed that a panel of metastasis-related genes were attenuated in PAGE4 overexpressing cells. In support, higher expression of PAGE4 predicted a better DFS of PCa in TCGA dataset, adhering to its inhibitory role of tumor aggressiveness. This is consistent with our previous finding that PAGE4 mRNA level was among markers correlated with a good prognosis of PCa [14]. Additionally, the previous finding that PAGE4 protein was detected more often in localized PCa than metastatic cancer highlights again the reverse correlation between PAGE4 expression and cancer aggressive phenotype [10]. Intriguingly, a recent elegant study links PAGE4 to the dynamic androgen-dependence and speculates that PAGE4 interacts with particular kinase suppresses AR hyperactivity and therefore makes cells sensitive to androgen deprivation (ADT) treatment [13], which may certainly lead to longer DFS. However, given that many metastatic PCa that are lack of PAGE4 expression are sensitive to ADT initially, it still could not be excluded that PAGE4 impacts cancer aggressiveness beyond ADT sensitivity. Thus, it is possible that PAGE4 blocks the development of aggressive PCa through attenuating the cell damage caused by oxidative stress which exists in the tumor microenvironment. To this rate, PAGE4 expression in PCa cells is potentially to be a predictive biomarker for good cancer prognosis, although it might promote tumor growth in primary site.

In consistent with our previous finding that PAGE4 is a stress-response protein [10], we here confirmed that PAGE4 expression was remarkably induced by ROS stimuli not only in cell models but also in xenografted tumor tissues. Notably, both endogenous PAGE4 expression and exogenously transfected PAGE4 construct can be induced by H_2_O_2_. This phenomenon was also noticed in our previous study, in which exogenously expressed PAGE4 was increased after treating cells with TNF- α that is a typical inflammatory chemokine [10]. These results consistently indicate that a post-transcriptional regulation may play an important role in up-regulating the expression of PAGE4 in response to stress stimuli.

Interestingly, PAGE4 has been shown to be interacted with two kinases, HIPK1 and CLK2, and leads to opposing functions. Particularly, CLK2 hyperphosphorylates PAGE4 resulting in attenuated function and likely rapid degradation [13]. In consistent with this result, we here found that co-expression of CLK2 with PAGE4 eliminated the effect of PAGE4 overexpression alone on reducing ROS production under oxidative stress. Notably, the inhibitory effect of CLK2 on PAGE4 function is similar in two PCa cells with different AR activity. However, the impact of HIPK1 on PAGE4 function is largely cell type-specific, namely enhancing PAGE4 protection on ROS in LNCaP cells that express functional AR but attenuating PAGE4 function in DU145 cells, in which AR is silenced. Given that it has been shown that HIPK1-PAGE4 interaction may affect AR activity in PCa cells [13], it is not surprise to observe that HIPK1 impacts PAGE4 activity in an AR-related manner, although many other differences in two cells may also potentially impact the interaction between PAGE4 and HIPK1. For example, HIPK1 has been shown to be activated by MAPK pathway and involved in ROS-induced cell death [56–58], while MAPK proteins associated with the HIPK1were more often mutated in DU145 but not in LNCaP cells [59]. Clearly, the interaction between PAGE4 and its catalysts under ROS stress should be further investigated.

In the present study, we found that PAGE4 significantly protected cell from DNA damage and cell death caused by ROS stress in cell models. The GSEA enrichment of DEGs also confirmed the protective effect of PAGE4 on DNA repair genes. Additionally, PAGE4 was found to promote tumor growth in vivo. It may be expected that cells suffer a high level of oxidative stress when growing in vivo, and ROS inhibition may reduce cell death caused by ROS and promote tumor growing. However, when we used NAC to neutralize ROS in the body of mice, the growth of xenografted tumors was likely to be impeded. To test, we found that NAC treatment significantly inhibited the expression of PAGE4 in tumor tissues. The decreased expression of PAGE4 following NAC treatment might partly explain why the growth of tumor was impeded but not promoted in this group, because PAGE4 was highly forced expressed in the tumors in the present study and its effect on protecting cell death and promoting cell survival is overwhelming compared to the effect of endogenous ROS on inducing cell death. Therefore, it is likely that the decreased expression of PAGE4 caused by ROS inhibition results in less cell survival, which is proposed to be more profound than the direct effect of ROS inhibition on reducing cell death. Consequently, the net result of NAC treatment on PAGE4 overexpressing-tumors would be the less cell survival, namely an impeded tumor growing. By all means, it is reasonable to speculate that the artificially enforced overexpression of PAGE4 in xenografted tumor would not completely mimic the pathological function of PAGE4 that is endogenously induced by microenvironmental ROS. In any rate, in this in vivo experiment, we observed the direct effect of PAGE4 overexpression on tumor survival, and we also confirmed that lowering ROS level in tumor tissue indeed decreased the expression of PAGE4, which nicely agrees with the stress-response role of PAGE4 to ROS.

In exploring the context of PAGE4 regulation on cell fate, RNA sequencing was conducted. We found a likely activation of MAPK pathway in PAGE4 overexpressing cell lines when they experienced ROS stress. It has been known that the MAPK signalling pathway regulates cellular process in varied aspects including cell proliferation, differentiation, mitosis, cell survival, and apoptosis [60]. Upregulation of JNK, a downstream gene in MAPK pathway, can cause apoptosis [43, 61]. Previous study showed that PAGE4 may interact with c-JUN and activate MARK pathway [20]. In the present study, MAPK/JNK pathway was activated after H_2_O_2_ treatment, while this activation was slightly lower in PAGE4 overexpressing cells than empty vector-transfected cell lines. This result suggests an attenuated apoptosis-inducing effect of ROS by PAGE4 overexpression. On the other hand, while activating of ERK1/2 is known to promote cell survival under ROS stress [43, 44], here we found that ERK1/2 was greatly activated by PAGE4 overexpression. Supportively, activated ERK1/2 was found to be largely colocalized with PAGE4 in human prostate tissue samples. These results strongly support the promoting effect of PAGE4 on cell survival. Further study should be warranted on the potential interaction between PAGE4 and ERK1/2 activation.

## Conclusions

In conclusion, the present study demonstrates the protective effects of PAGE4 in prostate cancer cells when exposed to oxidative stress_._ PAGE4 functions potentially through attenuating the phosphorylation of JNK to reduce apoptosis, and enhancing the phosphorylation of ERK to help cells survive. Clinically, the protection of PAGE4 on ROS stress may be translated into the less harmful DNA damage induced by ROS and therefore resulting in less chance to accumulate mutations to develop an aggressive cancer. Thus, a high expression of PAGE4 in PCa tissues may serve as a potential biomarker for better cancer prognosis.

## Additional files

Additional file 1: **Table S1.** Primers for Q-RT-PCR that were used in this article. (XLS 43 kb)

Additional file 2: **Figure S1.** ROS analysis in cells after H_2_O_2_ stimulate. (PDF 78 kb)

Additional file 3: **Figure S2.** Mice weight during the experiment. (PDF 75 kb)

Additional file 4: **Table S2.** 425 shared DEGs in 22RV1 and DU145 cell lines. (XLS 90 kb)

Additional file 5: **Figure S3.** PAGE4 shows low tumor malignancy. **a** Heat map for several prostate cancer malignancy related genes. **b** Disease-free survival analysis of PAGE4 in TCGA dataset. (PDF 91 kb)

## Abbreviations

ACTB: Actin Beta
BMP4: Bone morphogenetic protein 4
BPH: Benign prostatic hyperplasia
CRPC: Castration resistant prostate cancer
CTA: Cancer testis antigen
DEG: Differentially expressed genes
DOX: Doxycycline
ERK: Extracellular signal-regulated kinase
GSEA: Gene Set Enrichment Analysis
HIPK1: Homeodomain-interacting protein kinase 1
IDP: Intrinsic disorder protein
JNK: JUN N-Terminal Kinase
MAP2K4: Mitogen-Activated Protein Kinase Kinase 4
MAPK: Mitogen-activated protein kinase
NAC: N-Acetyl-L-cysteine
PAGE4: Prostate Associate Gene 4
PCa: Prostate cancer
PCR: Polymerase Chain Reaction
PI: Propidine iodide
ROS: Reactive oxygen species
SCID: Severe combined immunodeficiency
SEM: Standard Error of Mean
SOD1: Superoxide Dismutase 1
TGF-β1: Transforming growth factor beta 1
TNFα: Tumor Necrosis Factor
