# Supplementary figures and images for: PAGE4 promotes prostate cancer cells survive under oxidative stress through modulating MAPK/JNK/ERK pathway
Source: J Exp Clin Cancer Res. 2019 Jan 18;38:24. doi: 10.1186/s13046-019-1032-3 (PMC6339303; doi:10.1186/s13046-019-1032-3)

**a**

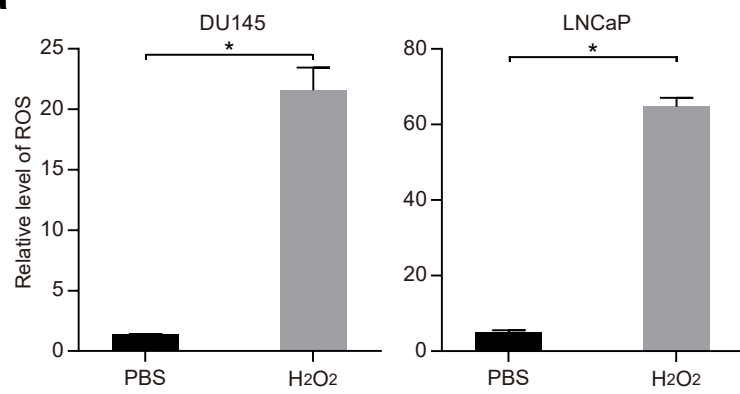

Supplement: Supplementary file 2 — Figure S1. ROS analysis in cells after H2O2 stimulate. (PDF 78 kb) [file 13046_2019_1032_MOESM2_ESM.pdf]

**a**

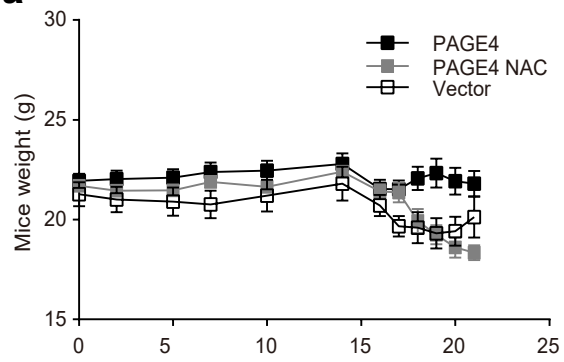

Supplement: Supplementary file 3 — Figure S2. Mice weight during the experiment. (PDF 75 kb) [file 13046_2019_1032_MOESM3_ESM.pdf]

**a**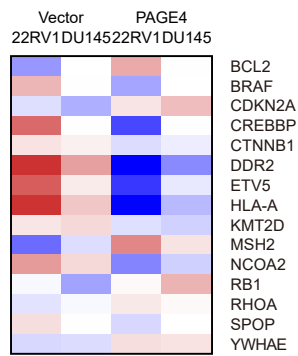**b**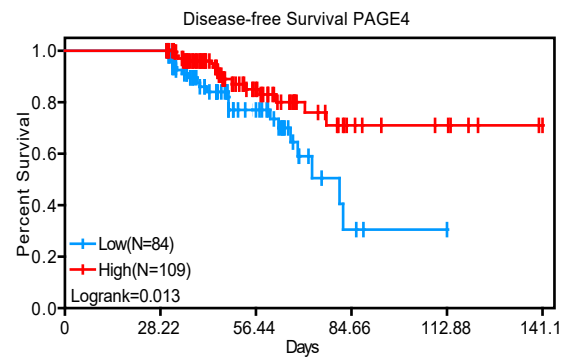

Supplement: Supplementary file 5 — Figure S3. PAGE4 shows low tumor malignancy. a Heat map for several prostate cancer malignancy related genes. b Disease-free survival analysis of PAGE4 in TCGA dataset. (PDF 91 kb) [file 13046_2019_1032_MOESM5_ESM.pdf]
